# The Oral Bacterial Communities of Children with Well-Controlled HIV Infection and without HIV Infection

**DOI:** 10.1371/journal.pone.0131615

**Published:** 2015-07-06

**Authors:** Brittany E. Goldberg, Emmanuel F. Mongodin, Cheron E. Jones, Michelle Chung, Claire M. Fraser, Anupama Tate, Steven L. Zeichner

**Affiliations:** 1 Division of Pediatric Infectious Diseases, Children’s National Medical Center, Washington, DC, United States of America; 2 Institute for Genome Sciences, University of Maryland School of Medicine, Baltimore, Maryland, United States of America; 3 Department of Microbiology and Immunology, University of Maryland School of Medicine, Baltimore, Maryland, United States of America; 4 Division of Pediatric Dentistry, Children’s National Medical Center, Washington, DC, United States of America; 5 Department of Medicine, University of Maryland School of Medicine, Baltimore, Maryland, United States of America; 6 Center for Cancer and Immunology Research, Children’s Research Institute, Children’s National Medical Center, Washington, DC, United States of America; 7 Departments of Pediatrics and Microbiology, Immunology, and Tropical Medicine, George Washington University School of Medicine, Washington, DC, United States of America; LSU Health Sciences Center School of Dentistry, UNITED STATES

## Abstract

The oral microbial community (microbiota) plays a critical role in human health and disease. Alterations in the oral microbiota may be associated with disorders such as gingivitis, periodontitis, childhood caries, alveolar osteitis, oral candidiasis and endodontic infections. In the immunosuppressed population, the spectrum of potential oral disease is even broader, encompassing candidiasis, necrotizing gingivitis, parotid gland enlargement, Kaposi’s sarcoma, oral warts and other diseases. Here, we used 454 pyrosequencing of bacterial 16S rRNA genes to examine the oral microbiome of saliva, mucosal and tooth samples from HIV-positive and negative children. Patient demographics and clinical characteristics were collected from a cross-section of patients undergoing routine dental care. Multiple specimens from different sampling sites in the mouth were collected for each patient. The goal of the study was to observe the potential diversity of the oral microbiota among individual patients, sample locations, HIV status and various dental characteristics. We found that there were significant differences in the microbiome among the enrolled patients, and between sampling locations. The analysis was complicated by uneven enrollment in the patient cohorts, with only five HIV-negative patients enrolled in the study and by the rapid improvement in the health of HIV-infected children between the time the study was conceived and completed. The generally good oral health of the HIV-negative patients limited the number of dental plaque samples that could be collected. We did not identify significant differences between well-controlled HIV-positive patients and HIV-negative controls, suggesting that well-controlled HIV-positive patients essentially harbor similar oral flora compared to patients without HIV. Nor were significant differences in the oral microbiota identified between different teeth or with different dental characteristics. Additional studies are needed to better characterize the oral microbiome in children and those with poorly-controlled HIV infections.

## Introduction

During development, the oral microbial community changes dramatically, from the sterile or minimal environment present in prenatal life, to the large microbial exposures that accompany birth, to the environment that exists as the infant begins to feed and interact with the environment. As children grow and mature, additional changes in the oral microbial community accompany the eruption of the primary teeth, and the oral microbial community develops into the community that exists in association with mixed and finally adult dentition. The differences in the oral microbial communities that accompany dental development are clearly reflected in clinically appreciated changes. For example, the prevalence of plaque and the quality of plaque differs significantly in children with primary and permanent dentition [1], with for example, some species associated with periodontal disease rarely observed in children with only primary dentition [2]. The prevalence of caries also changes substantially during development. Some factors that potentially influence the oral microbiota include the hormonal changes associated with puberty which can affect host responses and the oral microbiota, and are thought to play a role in the increase in periodontal disease seen in adolescents and adults [3].

The oral cavity includes an impressive variety of micro-environments. Some areas are more aerobic, such as the tongue and buccal surfaces, other areas are more anaerobic, such as subgingival plaques, while some areas are exposed to differing amounts of salivary flow. Studies using both conventional and molecular techniques have shown that there are substantial differences in the microbial communities that inhabit these different microenvironments. Culture-independent approaches using next-generation sequencing have also demonstrated variety in the microbiota between different sites within the oral cavity, particularly between mucosal and dental samples [4–7].

A relatively small number of studies have explored the changes in the oral microbiota that accompany dental development. Most of these studies have used conventional, culture-based techniques [8, 9] or molecular techniques designed to detect only specific species [2, 10–12], many with known associations with particular diseases, like caries. A study of 74 children using 454 pyrosequencing on saliva samples examined the changes associated with the stages of oral development [13], in which the relative abundances of bacterial phyla were found to change compared to adult dentition.

With the increase in culture-independent studies of microbial communities, much interest has focused on the oral cavity [14–20]. Resources such as the Human Oral Microbiome Database (HOMD) project have arisen to provide the scientific community with a comprehensive repository of genomic, phylogenetic, phenotypic, clinical, and bibliographic information of the prokaryote species identified in the human oral cavity. As next-generation sequencing technology has become more accessible, small-scale studies have been conducted to evaluate the oral microbiota of healthy adult individuals [4, 5]. These studies have found a certain degree of overlap between bacterial species in healthy adult volunteers, leading to the proposal of a common ‘core’ oral microbiota [4, 7, 15]. Similarly, a core microbiota associated with periodontal disease has also been described using whole-genome sequencing [6].

While many studies have been directed at adult samples and, in particular, the microbiota associated with particular oral diseases, like caries or periodontitis [10, 18, 21], only a few studies have focused on children. In some pediatric studies, culture-independent studies using 16S rRNA gene sequences have been employed, but many of these have used older sequencing approaches that have difficulty providing sufficient depth of coverage to identify potentially low abundance species [11, 20]. These studies, however, showed clear development-dependent changes in different components of the oral microbial community. For example, one study found that in plaque, *Prevotella nigrescens* was found more frequently and its prevalence increases with increasing age; *Eikenella corrodens* and *Campylobacter rectus* were detected in plaque and sometimes in saliva, and *Tannerella forsythensis* was sometimes seen in saliva. Some species, like *Prevotella intermedia*, *Porphyromonas gingivalis*, and *P*. *nigrescens*, showed age-related prevalence changes; the number of subjects with no detectable target species dropped from 40% at 3 y to 0% at 11 y, demonstrating strong age-dependent changes in oral microbiota [22].

Some studies have characterized the flora associated with specific pediatric oral diseases, and have identified particular agents associated with pediatric oral disorders, such as caries [23], but these studies also tend to focus on microbes identified through conventional culture techniques. It appears that HIV infection also impacts the oral microbiota, as demonstrated by a small study comparing HIV positive patients to negative controls using microarray assays of dorsal tongue samples [24].

We initiated this study to develop approaches for the study of the pediatric oral microbiota, to observe the potential diversity of the oral microbiota among individual patients, sample locations, HIV status, and dental characteristics to provide a background for future studies of the oral microbiota in children with immunosuppressive disorders, including HIV disease, which has a distinct set of associated oral disorders, including those with a substantially higher prevalence in children [25–29]. While increasing interest has focused on characterizing and understanding the oral microbiota [16], less work has been done to study the microbiota in a developmental context. We conducted a cross-sectional study of normal children, seen for routine dental care, including children with primary, mixed, and adult dentition, and including periodontal, lingual, buccal, and saliva samples. Only five HIV-negative patients consented to enroll in the study. However, we found significant changes in the composition of operational taxonomic units (OTUs), a measure of bacterial species, between primary and adult dentition. It should be noted that HIV-negative patients had generally good dentition which limited the number of dental plaque samples that could be attained. Additionally, limited enrollment and a substantial decrease in the population of highly immunosuppressed pediatric HIV patients as the study got underway precluded a comparison of immunocompromised HIV-positive patients to HIV-negative or well-controlled HIV-positive patients.

## Materials and Methods

### Subjects and Samples

The oral health status of all individuals was determined by or under the supervision of the dental investigator (A.T.). Entry criteria included patients presenting to the Dental Clinic for routine care for which, in the opinion of the dentist and attending physician, collection of samples represented no more than a minimal risk to the patient. Specific exclusion criteria included a patient with a disease for which the American Heart Association guidelines recommended antibiotic prophylaxis. The patients underwent a comprehensive dental exam, with the state of dentition and any clinical dental findings captured in a structured data capture instrument. A comprehensive medical history was also obtained, including any history of immunosuppressive conditions and medications, particularly those such as antibiotics that could potentially affect the oral flora. Data concerning race and ethnicity (Table 1), and other factors that could potentially affect the oral flora, such as chewing gum use were also collected. Cariogenic exposures (that is, foods with a propensity to cause dental caries, as classified by the American Dental Hygienists' Association (http://www.adha.org/ce-course-7) were based upon dietary recall from questioning the patients’ parents (Tables 2 and 3). The number of cariogenic exposures, classified as 0 to 5, or more than 5 are listed in Table 2. Variables with more than three patients in each arm were chosen for additional statistical analysis. Parental assistance was defined as adult supervision with tooth brushing or home dental care, and removable appliances included intra-oral dental devices.

**Table 1:** Patient Demographics^a^.

Patient ID	Age (yrs)	Gender	Race	HIV Status	Past Medical History	Plaque Score	Medications	Oral Pathology	CD4 Percent (%)	CD4 Absolute Count (cells/mm^3^)	Viral Load (copies/mL)
1	13	Male	African American	HIV/B1	None	Poor	LPV/r, TDF, ZDV	None	40	770	Undetectable
2	13	Female	African American	HIV/C3	None	Fair	d4T, NFV, DDI,	None	60	1200	<100
3	12	Male	African American	HIV	None	Poor	LPV/r, 3TC, ZDV	LGE	30	530	Undetectable
4	10	Female	African American	HIV/B3	None	Poor	ABC/3TC, EFV, TMP/SMX	None	30	370	<100
6	15	Male	African American	HIV/B3	None	Fair	LPV/r, TDF, d4T	None	30	350	<100
7	15	Female	African American	HIV/B2	None	Fair	d4T, ABC, LPV/r,	None	20	620	<150
8	13	Male	African American	HIV	Asthma	Fair	Unknown	Unknown	Unknown	Unknown	Unknown
9	14	Female	Native American	HIV/N1	None	Poor	NVP, ABC, d4T	None	36	1930	Undetectable
11	17	Female	African American	HIV/C3	None	Fair	EFV/FTC/TDF,	None	40	760	Undetectable
12	5	Female	African American	HIV	None	Fair	ZDV, 3TC, NVP	None	40	1970	Undetectable
13	12	Female	African American	HIV/B2	None	Fair		None	20	380	<1500
10	10	Male	African American	Neg.	None	Fair	None	None	N/A	N/A	N/A
14	12	Male	White	Neg.	None	Fair	None	None	N/A	N/A	N/A
15	16	Female	White	Neg.	None	Good	None	None	N/A	N/A	N/A
16	8	Female	African American	Neg.	None	Fair	None	None	N/A	N/A	N/A
17	4	Female	African American	Neg.	None	Good	None	None	N/A	N/A	N/A

^a^ Table 1 contains demographic information for all patients with sequenced samples. No additional treatment information was available for patient 8. Ages, viral loads and CD4 values were rounded to two significant digits to maintain patient privacy.

3TC–lamivudine, ABC–abacavir, AZT—d4T = stavudine, ddI–didanosine, EFV–efavirenz, FTC–emtricitabine, LPV/r–lopinavir/ritonavir, NFV–nelfinavir, NVP–nevirapine, TDF–tenofovir, ZDV–zidovudine, SMZ-TMP–sulfamethoxazole and trimethoprim, LGE–linear gingival erythema.

**Table 2:** Dental Characteristics^a^.

	HIV-Positive Patients n = 8		HIV-Negative Patients n = 5	
	Yes	No^b^	Yes	No
**Parental Assistance**	0	8	2	3
**Mouthwash**	2	6	4	1
**Fluoride Water**	8	0	5	0
**Toothpaste w/Fluoride**	4	4	5	0
**Removable Appliance**	1	7	1	4
**Probiotics**	5	3	4	1
**Chewing Gum**	6	2	4	1
**0 to 5 Cariogenic Exposures**	6	2	1	4

^a^ Patient dental characteristics were collected for each enrolled patient. No data was available for three HIV-positive patients.

^**b**^ The indicator “No” for the entry “0 to 5 Cariogenic Exposures” indicates that the patient had more than 5 cariogenic exposures.

**Table 3:** Daily Dental Care.

	HIV-Positive Patients n = 8			HIV-Negative Patients n = 5		
	**0x**	**1x**	**≥2x**	**0x**	**1x**	**≥2x**
**Daily Brushing Frequency**	0	4	4	0	3	2
	**Never**	**Sometimes**	**Daily**	**Never**	**Sometimes**	**Daily**
**Flossing Frequency**	6	1	1	2	2	1

The dental exam included a full-mouth clinical examination with inspection of the teeth (assessment of deciduous and adult teeth, and restored and unrestored carious lesions and method of restoration), oral mucosa, and periodontal tissues. The clinical impression of the oral exam was recorded and abstracted in a structured format for inclusion with the clinical metadata for each patient, which was entered into a database. Periodontal examination included assessments of clinical signs of inflammation (redness, swelling, or bleeding on probing (BOP)), and of the presence or absence and extent of gingivitis or periodontitis. When in the full permanent dentition, probing depths (PD) and clinical attachment loss (CAL) measurements were recorded on 4 sites/tooth (MB, B, DB, L) of all teeth except third molars using a calibrated North Carolina periodontal probe. Sites were considered periodontally healthy if there was no BOP or other signs of clinical inflammation combined with shallow PD (≤ 4 mm) and minimal CAL (≤ 2 mm). Subjects were considered periodontally healthy if on a full-mouth basis there is little or no BOP (≤ 2% of sites), the average PD is ≤ 3 mm, and mean CAL is ≤ 0.6 mm. Details of the periodontal data from sites were recorded and abstracted as clinical metadata. From each individual in the full permanent dentition, up to 26 oral specimens could have been collected. Separate dental plaque specimens were taken with sterile curettes from supragingival and subgingival surfaces of 7 target teeth (#3, 9, 12, 19, 25, 28, and 30). In the primary or mixed dentition, 10 oral specimens were collected. In many cases, there was no plaque on the target tooth. Separate dental plaque specimens were taken with sterile curettes from supragingival surfaces of 5 target teeth (#A, T, E, P, I) if present. Although the protocol allowed for collection of subgingival samples, all samples were supragingival due to the generally good dental health of the enrolled patients. In cases where a target tooth is missing or otherwise not suitable for sampling, a nearby tooth was used as a substitute. However, many nearby teeth also lacked plaque or were otherwise unsuitable and could not be sampled. Sterile plastic spatulas were used to collect soft tissue scrapings from the keratinized buccal gingiva adjacent to each of the target teeth, dorsal and ventral surfaces of the tongue, and buccal mucosa. The dental protocol contained provisions to obtain hard palate plaque samples, but none were observed during enrollment. The final sample consisted of whole saliva that was expectorated into a test tube. Specimens were suspended in 200 μl of sterile phosphate buffered saline (PBS) in screw-cap vials. Specimens were frozen immediately, and kept at -80°C before further processing. The study was approved by the Children’s National Medical Center Institutional Review Board and patients were enrolled in the study following signed informed consent by the parent/guardian and assent where appropriate.

### DNA Extraction and 16S rDNA Amplification

Total genomic DNA was extracted using a protocol developed at the University of Maryland School of Medicine—Institute for Genome Sciences and previously described [30]. Briefly, samples were thawed on ice, incubated in an enzymatic cocktail containing lysozyme, mutanolysin, proteinase K and lysostaphin, after which the microbial cells were lysed using bead beating with silica beads (Lysing Matrix B, MP Biomedicals) with the FastPrep instrument (MBio, Santa Ana, CA). The DNA was then further extracted and purified using the Zymo Fecal DNA kit (Zymogen).

The variable regions V1–V3 of the 16S rRNA gene were PCR amplified using barcoded 27F and 338R 16S primers, as described previously [30]. The ideal primers for 16S PCR are still under investigation. Negative controls without a template were included for each barcoded primer pair. The presence of PCR amplicons was then confirmed by gel electrophoresis on a 2% agarose gel and staining with ethidium bromide. PCR products were quantified using the Quant-iT PicoGreen dsDNA assay, and equimolar amounts (100 ng) of PCR amplicons were pooled prior to pyrosequencing [30]. This 16S PCR amplicon pool was sequenced by 454 FLX Titanium sequencing technology using 454 Life Sciences primer A by the Genomics Resource Center at the Institute for Genome Sciences, University of Maryland School of Medicine, using protocols recommended by the manufacturer as amended by the Center.

### Sequence Analysis

Sequences were analyzed using the open-source computational package Qiime 1.8.0 using the QIIME implementation of USEARCH for quality filtering and chimera check, and alignment against the SILVA rRNA database, release 111. Each 454 pool was denoised to remove erroneous sequences, prior to OTU assignment and alignment. Data were clustered into OTUs using the open reference OTU picking strategy. Additional filtering was performed to retain OTUs present in greater than 25% of all samples[31–33]. All sequence files were uploaded into the NCBI short read archive (SRA) under accession number SRP056328.

### Statistical Methods

The statistical analysis was conducted using Qiime version 1.8 and the open-source statistical program R. Alpha (Shannon, Simpson and Chao1) and beta (Brey-Curtis) diversity scores were calculated through QIIME. These diversity score values and the OTU abundance data was loaded as a tab-delineated text file into R. Patient oral microbiome data was examined between the individual patients, specimen location, tooth age (primary versus permanent), HIV status, and dental characteristics. Only dental characteristics with more than three patients per variable were chosen for additional analysis, and certain dental characteristics, such as diet, lacked sufficient diversity to warrant further statistical analysis. The R software package was used to calculate ANOVA scores and Welsh two-tailed t-tests. NMDS and PCoA coordinates were also calculated using the R software package Vegan, and graphs were generated in Microsoft Excel.

## Results

We conducted a cross-sectional single time point study, enrolling sixteen children from age 4 to 16 years and varying stages of dental development, and employing a convenience sampling strategy of children attending the Dental Clinic at the Children’s National Medical Center (CNMC). The demographic characteristics of the patients are summarized in Table 1. Of the sixteen enrolled patients, eleven were HIV infected with excellent viral suppression. Descriptive information regarding dental practices is reported in Tables 2 and 3.

A total of 144 samples were collected for DNA analysis. rRNA gene sequences were PCR amplified and sequenced using 454 Titanium pyrosequencing. Of these 144 samples, 4 failed to amplify after several PCR attempts and were eliminated from the analysis. The remaining samples produced 1,629,446 non-chimeric sequences clustered in a total of 2,641 operational taxonomic units (OTUs) using a cutoff sequence identity of 97% (roughly equivalent to species-level OTU clustering). The average number of sequences per specimens was approximately 22,000, but includes chimeric sequences. Additional filtering to remove low abundance OTUs left 851 OTUs in the final analysis data set.

**Fig 1:**
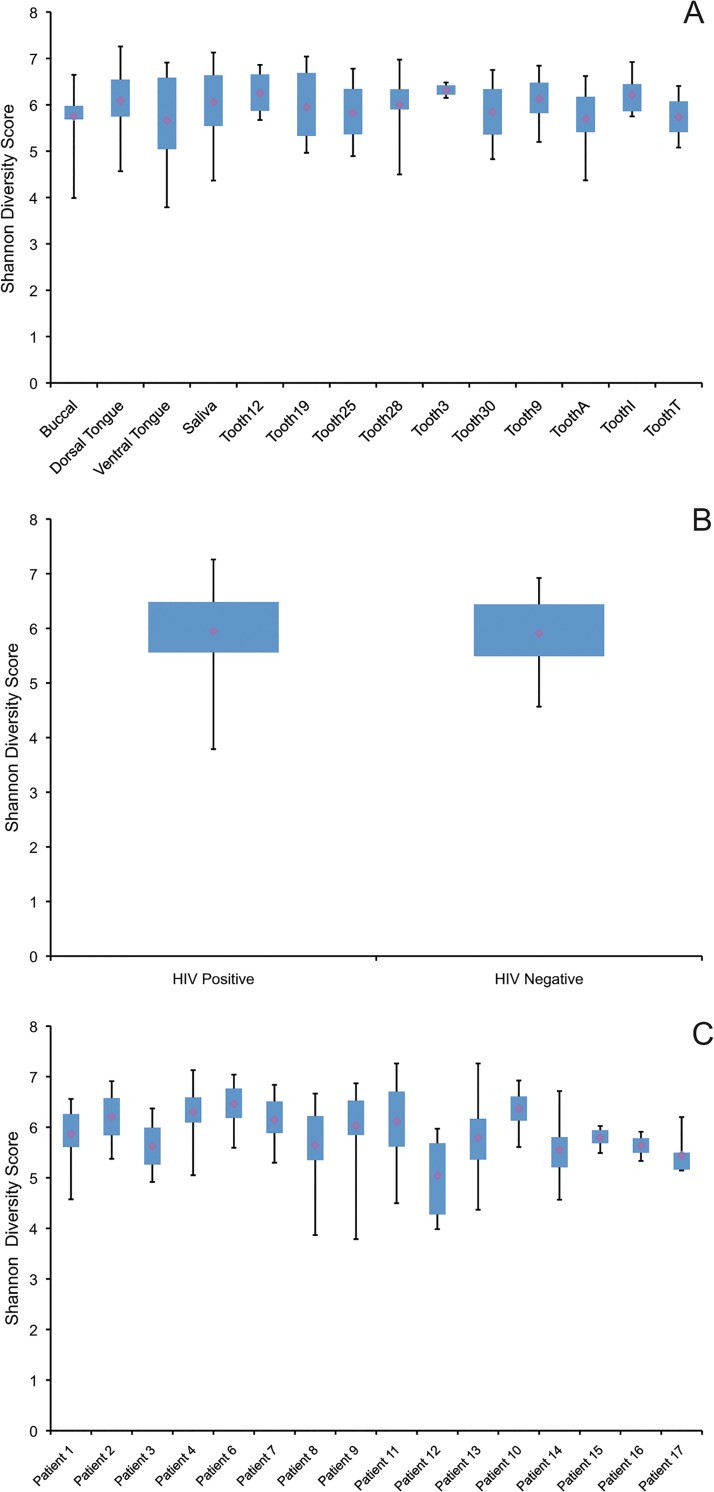
Shannon Diversity Scores. Boxplots were generated from Shannon diversity scores with quartiles represented in whisker plots and the mean identified by the central point. The Shannon score, which is a measure of species diversity within a sample, is compared between sample locations in Panel A. All Shannon scores for each location, patient or HIV-status are contained in the individual boxplots. Panel B demonstrates that minimal differences were observed between the HIV positive and HIV negative patients. The range of Shannon scores between patients is shown in Panel C. Most patients had scores greater than 5.6. The sole exception was patient 12, who was the only patient under age five. All tooth samples from patient 12 were from primary teeth, and her average shannon score was decreased relative to the other patients with a value of 5.0. All tooth samples are supragingival.

Diversity (Shannon and Simpson) and richness (Chao1) indices were calculated for each sample; median values and standard deviations are provided by sample location and HIV status in Table 4. Both Simpson and Shannon diversity indices provide insights into species composition and relative abundance of bacterial communities. However, with the Shannon index, the "weight" of abundant species is reduced slightly relative to more rare species, whereas using the Simpson index the weight of rare species is reduced relatively more than that of more abundant species [34]. The Chao1 estimator scores the community richness, e.g. the total number of species present in a sample. Table 4 demonstrates that the mean diversity metrics and observed number of OTUs fell within a narrow range across the different sampling sites and patient populations. The total number of observed OTUs ranged from 792 to 851, mean Chao1 scores ranged from 476.66 to 560.24 mean Shannon scores ranged from 5.66 to 6.25 and mean Simpson scores ranged from 0.92 to 0.97. Mean Shannon values for each individual patient ranged between 5.04 and 6.46 (Fig 1C).

**Table 4:** Diversity and Richness Estimators^a^.

	N	Observed Number of OTUs	Chao1	Shannon	Simpson
Tooth9	11	848	519.39 (91.54)	6.13 (0.52)	0.96 (0.02)
Tooth12	8	847	552.70(65.77)	6.25 (0.48)	0.96 (0.01)
Tooth19	11	838	491.5(121.45)	5.95 (0.74)	0.95 (0.03)
Tooth25	11	832	476.66(87.06)	5.83 (0.63)	0.94 (0.04)
Tooth28	8	792	484.05(120.26)	6.00 (0.75)	0.95 (0.03)
Tooth30	11	842	538.68(63.56)	5.84 (0.67)	0.94 (0.04)
Tooth3	9	839	538.68(63.56)	6.31 (0.13)	0.97 (0.01)
Buccal	16	846	543.88(102.42)	5.75 (0.59)	0.94 (0.03)
Dorsal Tongue	15	850	560.24(107.22)	6.09 (0.72)	0.95 (0.02)
Ventral Tongue	15	848	523.08(112.49)	5.66 (1.03)	0.92 (0.07)
Saliva	14	847	547.28(132.53)	6.06 (0.76)	0.95 (0.03)
All HIV negative	25	851	495.00(100.34)	5.91 (0.60)	0.95 (0.04)
All HIV positive	115	849	534.9 (94.56)	5.95 (0.74)	0.95 (0.03)

^a^ Mean values of Chao1, Shannon and Simpson diversity indexes are shown for all samples with at least one OTU in greater than 25% of all samples and with an n of greater than 4. Standard deviation is shown in parentheses. Total number of samples and OTUs for each site is displayed.

**Fig 2:**
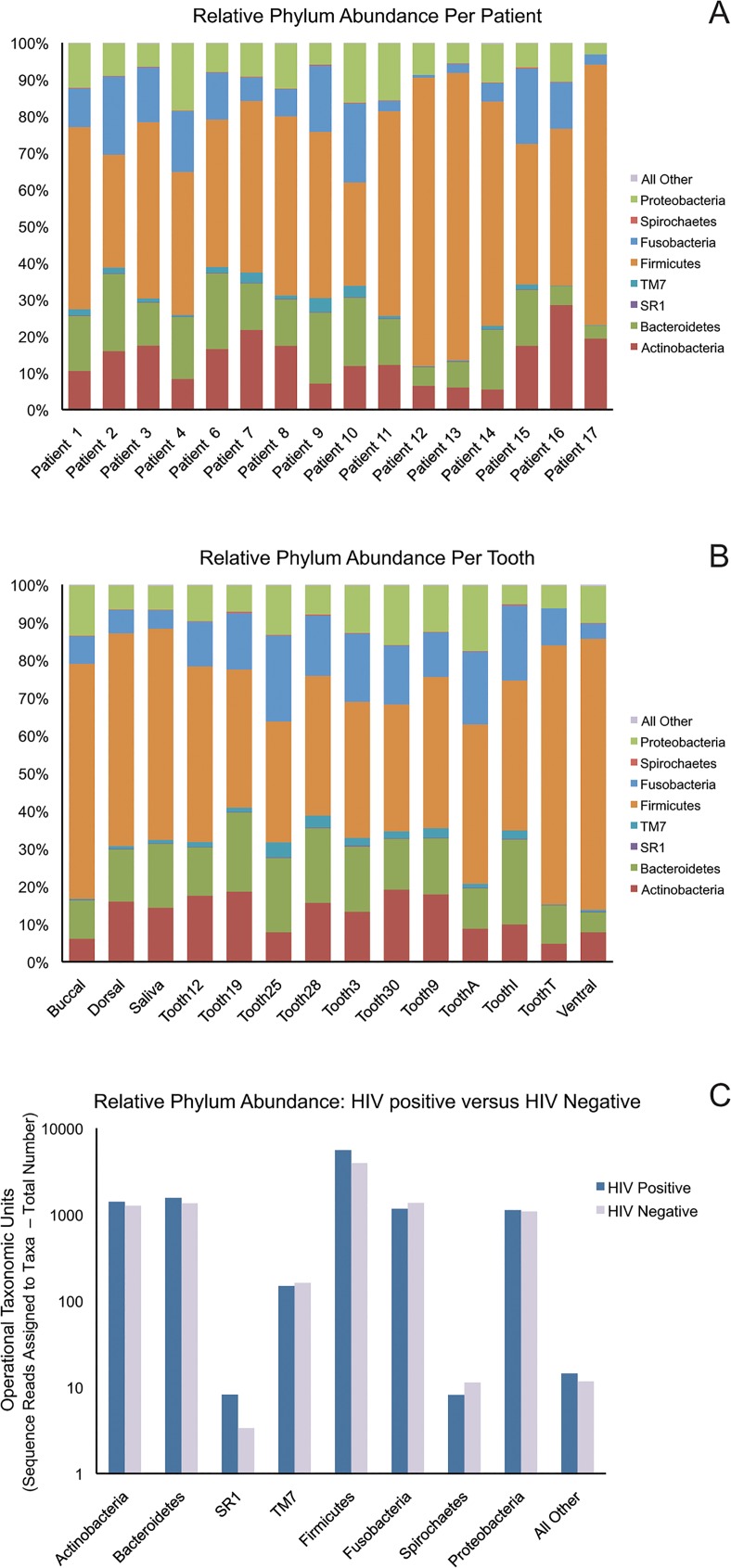
Relative Abundance Data at the Phylum level. All samples with OTUs identified to the phylum level were summarized as bar charts of relative abundance. Relative abundance varied by patient (Panel A) and sample site (Panel B). Panels A and B contain all samples available for each individual patient and sampling sites. The OTU count for each phyla from HIV positive and negative samples were summed in Panel C, demonstrating increased abundance of Firmicutes in HIV positive patients. All tooth samples are supragingival.

Relative phylum abundance data from each sample were analyzed as parts of three cohorts stratified by: (1) individual patients (2) sample location and (3) HIV status. Fig 2 contains relative abundance data for each cohort. Uneven enrollment between the oral sampling locations compromised comparison between patients and locations (Table 5). The total number of samples obtained for each patient was affected by their underlying plaque quantities. Some samples could not be obtained due to low plaque quantity. HIV patients averaged 10.5 samples per patient, while healthy patients averaged 5 samples per patient. Individual patients had variable proportions of different phyla, but it is unclear if differences in relative abundance are due to true inter-individual variability in the oral microbiota or sampling bias (Fig 2A). Generally, samples from saliva or mucosal surfaces appeared to have a higher relative abundance of Firmicutes phyla. Samples from teeth had a greater variety of different phyla, with phylum TM7 and Spirochaetes appearing in some samples (Fig 2B). The relative abundances of the phyla from tooth samples also appear more evenly proportioned, particularly when compared to the Firmicutes dominance of the mucosal surfaces. Between all HIV infected patients and all healthy patients, no statistically significant differences between the relative abundances of different phyla were appreciated. Firmicutes was the dominant phylum in both HIV-positive and HIV-negative patients, with Fusobacteria and Bacteroidetes accounting for the next most prevalent phyla (Fig 2C).

**Table 5:** Patient Sequencing Data^a^.

	Patient Identifier															
	1	2	3	4	6	7	8	9	10	11	12	13	14	15	16	17
**Saliva**	**X**		**X**	**X**	**X**	**X**	**X**	**X**		**X**	**X**	**X**	**X**	**X**	**X**	**X**
**Dorsal Tongue**	**X**	**X**	**X**	**X**	**X**	**X**	**X**	**X**	**X**	**X**	**X**	**X**	**X**	**X**		**X**
**Ventral Tongue**	**X**	**X**	**X**	**X**	**X**	**X**	**X**	**X**	**X**	**X**		**X**	**X**	**X**	**X**	**X**
**Buccal**	**X**	**X**	**X**	**X**	**X**	**X**	**X**	**X**	**X**	**X**	**X**	**X**	**X**	**X**	**X**	**X**
**Tooth 12**	**X**	**X**	**X**		**X**	**X**	**X**			**X**		**X**				
**Tooth 19**	**X**	**X**	**X**	**X**	**X**	**X**	**X**	**X**	**X**	**X**		**X**				
**Tooth 3**			**X**	**X**	**X**	**X**	**X**	**X**	**X**	**X**		**X**				
**Tooth 30**	**X**	**X**	**X**	**X**	**X**	**X**	**X**	**X**	**X**	**X**		**X**				
**Tooth 9**	**X**	**X**	**X**	**X**	**X**	**X**	**X**	**X**	**X**	**X**		**X**				
**Tooth 25**	**X**	**X**	**X**	**X**	**X**	**X**	**X**	**X**	**X**	**X**		**X**				
**Tooth 28**	**X**	**X**	**X**		**X**	**X**	**X**	**X**		**X**						
**Tooth P**											**X**					
**Tooth E**											**X**					
**Tooth T**				**X**							**X**					
**Tooth A**			**X**	**X**					**X**		**X**					
**Tooth I**								**X**	**X**		**X**					
**Total**	**10**	**9**	**12**	**11**	**11**	**11**	**11**	**11**	**10**	**11**	**8**	**10**	**4**	**4**	**3**	**4**

^a^ Sample was not uniform for each enrolled patient. Four samples did not provide sufficient DNA amplicons for sequencing. Permanent teeth include Tooth 12 (first premolar), 19 (first molar), 3 (first molar), 30 (first molar), 9(central incisor), 25 (central incisor) and 28 (first premolar). Primary teeth include Tooth P (central incisor), E (central incisor), T (second molar), A (second molar) and I (first molar). Anterior teeth include Tooth 9, 25, P and E. Posterior teeth include 3, 12, 19, 28, 30, A, T and I.

Shannon bacterial diversity scores were analyzed using a Welsh two-sample t-test and ANOVA (Table 6). The Shannon diversity scores were significantly different between individual patients (p = <0.05) and between HIV-infected patients (p = <0.05), but pairwise t-testing did not identify a specific patient responsible for these differences (Table 6). Patient 12 was the youngest subject enrolled and presented with only primary dentition at the time of sampling. All diversity scores for patient 12 were noticeability lower compared to the other patients (Fig 1C), although ANOVA testing did not identify a statistically significant difference, likely secondary to the small sample size. Most patients’ diversity scores ranged between 5.6 and 6.3. However, patient 12 had a notable decrease in species diversity, with a score of 5.0.

**Table 6:** Shannon Diversity Scores^a^.

	P-value (95% CI)
All Patients	**0.00019**
All HIV Patients	**0.00062**
All Samples	**0.019**
All Teeth	0.08
All Permanent Teeth	0.50
All Primary Teeth	0.34
Primary vs. Permanent Teeth	0.16 (-0.21,1.06)
Anterior vs. Posterior Teeth	0.37 (-0.49, 0.19)
HIV-Positive vs. HIV-Negative Patients	0.99 (-.028, 0.29)
CD4 Count less than 500	0.06 (-0.59, 0.01)
Plaque Score	0.40
Flossing Frequency	0.92
Mouthwash Use	0.13 (-0.06,0.48)
Probiotic Use	0.13 (-0.50, 0.06)

^a^ All Shannon diversity scores for each subject group was compared using two-tailed t-tests and ANOVA statistics.

**Fig 3:**
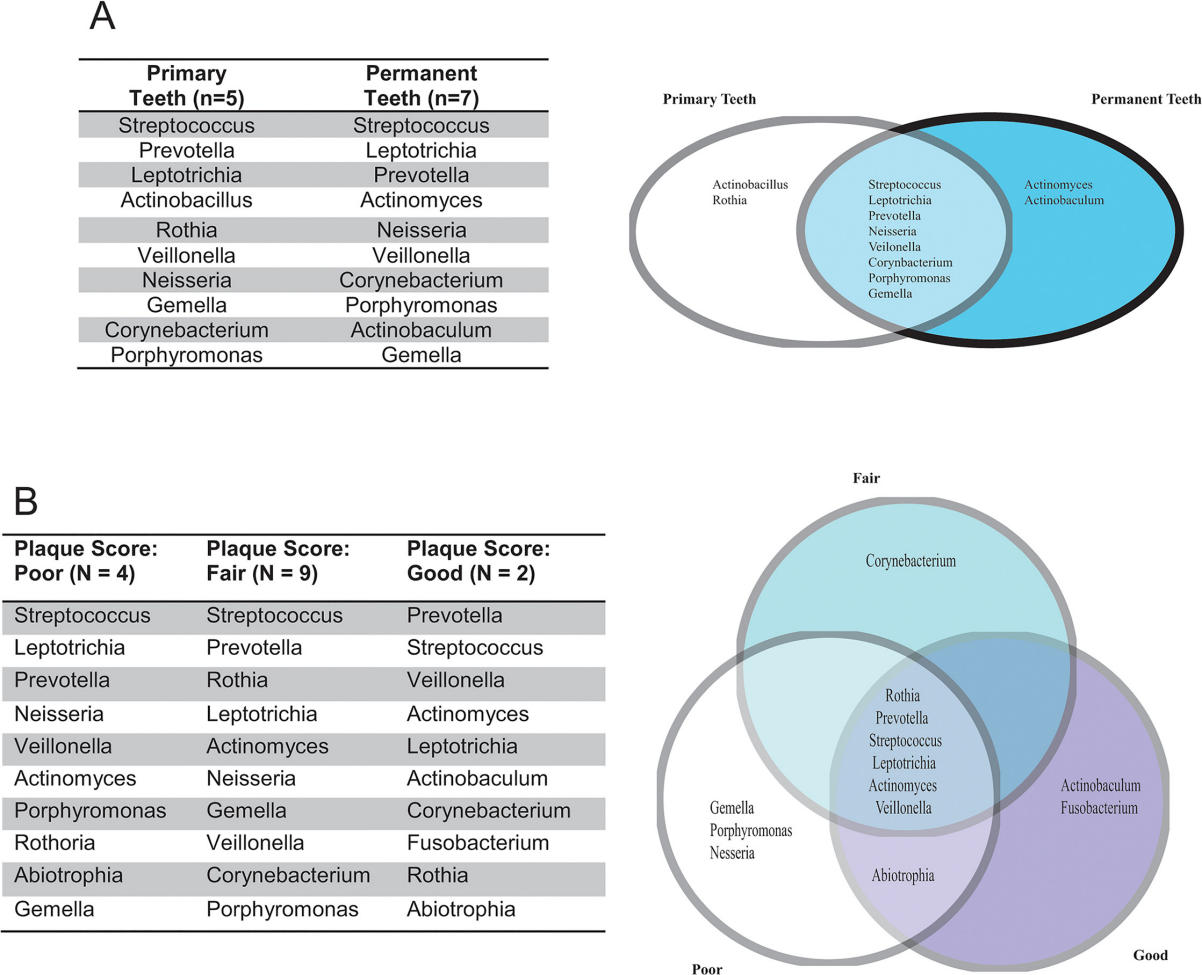
Most abundant OTUs. Relative OTU abundance was summed for primary versus permanent teeth and plaque score. Multiple OTUs may be generated for the same genera, due to the clustering method employed by QIIME. Unique genera consist of OTUs with identical genera assignments, which were summed for each patient group to provide a total abundance for each unique genus. The abundance data was normalized and ranked to identify the ten most abundant genera for each group. Genera identified in only primary or permanent teeth were present in both patient groups, but at lower relative abundance.

Although statistically significant differences were not appreciated, a comparison of relative abundances between unique genera in primary and permanent dentition revealed increased abundance of *Rothia* and *Actinobacillus* in primary teeth, while *Actinomyces* and *Actinobaculum* were more abundant in permanent teeth (Fig 3A). The clustering algorithm used by QIIME results in the generation of multiple OTUs with identical taxonomic assignments. When ranking the genera present in samples, all samples with an identical taxonomic assignment were combined. Unique genera refer to non-duplicated taxonomic assignments, for which the relative OTU abundance was summed and then ranked to produce Fig 3.

Significant differences between Shannon diversity scores in the various sampling locations were not observed for all teeth, between primary and permanent teeth or between anterior and posterior teeth (Table 6). Additional dental characteristics, including plaque score, flossing frequency, mouthwash use and probiotic use, were examined for their effects upon the relative abundance of OTUs, but were not found to be significantly different. However, significant differences were identified between the Shannon diversity scores of all samples, which included both tooth samples and mucosal samples. No particular sample type could be identified as the source of the difference. The sampling size differences were particularly marked between tooth and mucosal samples, which made comparison challenging. Although differences between plaques scores were not statistically significant, they were used as a marker of dental health to compare changes in the bacterial populations. The relative abundances of each unique OTU between the plaque scores were compared to identify changes in the bacterial population (Fig 3B). The genera *Actinomyces*, *Prevotella*, *Rothia*, *Streptococcus*, *Leptotrichia* and *Veillonella* were represented in all samples. The two patients who received a ‘Good’ plaque score had increased relative abundance of *Actinobaculum* and *Fusobacterium*. The patients with ‘Fair’ or ‘Poor’ plaque scores contained the genera *Gemella*, *Neisseria* and *Porphyromonas*.

**Fig 4:**
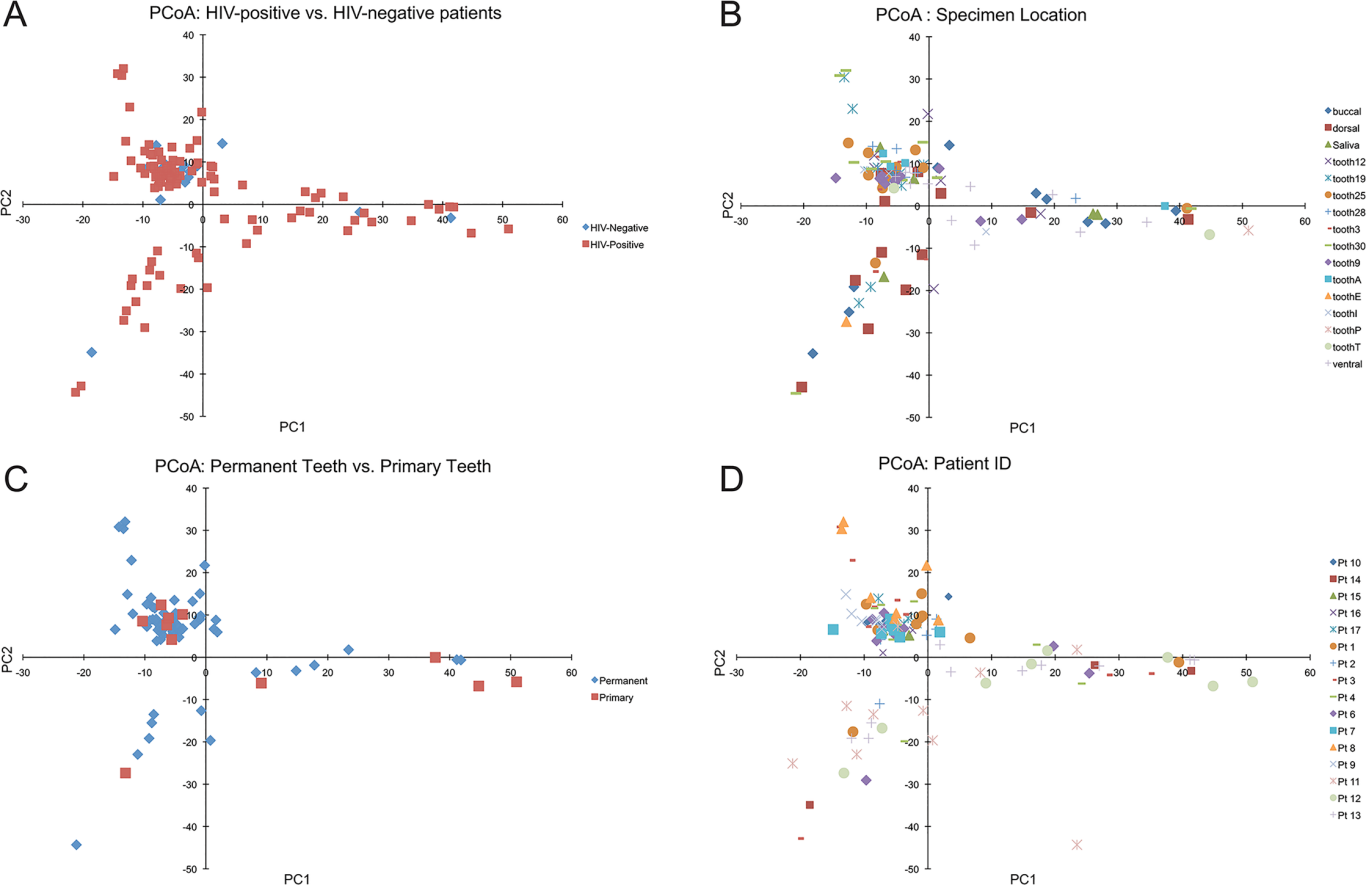
Principle Coordinate Plots. Principle coordinate plots were generated from OTU abundance data using the Vegan package in R. Panel A demonstrates coordiates from samples labeled by the HIV status. Panel B contains coordinates from samples labeled by the sample location. Panels C and D depict coordinates from samples labeled by dentition status and patient ID respectively.

**Fig 5:**
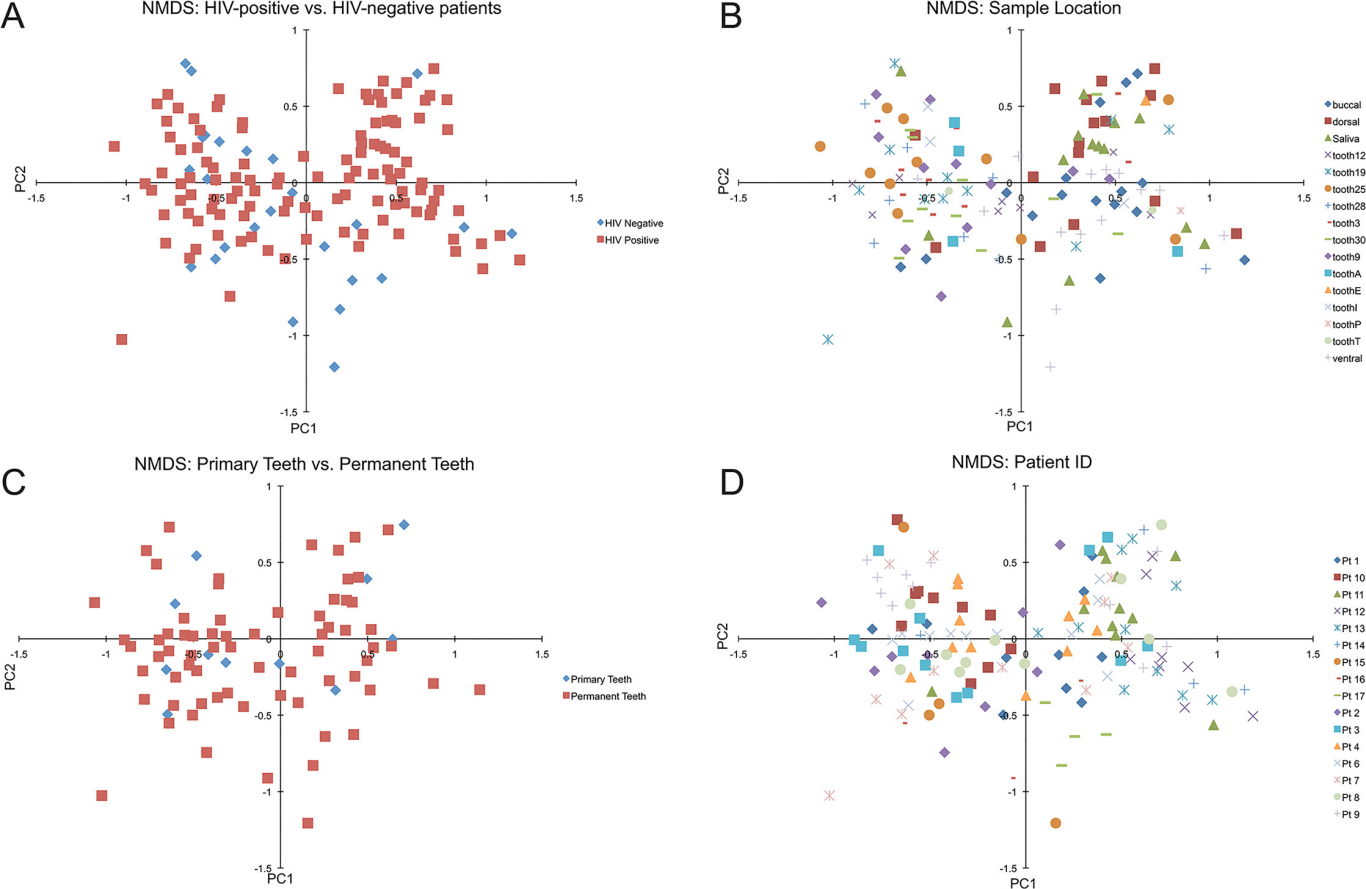
NMDS Coordinate Plots. NMDS plots were generated using OTU abundance data using the Vegan package in R. Panel A and B contain coordinates labeled by HIV status and sample location respectively, while Panel C and D contain coordinates labeled by dentition status and patient ID.

Fig 1 compares Shannon diversity scores between samples, HIV status and patients. Samples taken from teeth tended to have higher diversity scores, but teeth had fewer samples compared to saliva and mucosa (Fig 1A). Diversity between the HIV and healthy cohorts did not vary significantly (Fig 1B) with a p-value of 0.9898. Individual patients were also compared, as shown in Fig 1C, and discussed above. NMDS and Principle Coordinate plots were generated using OTU abundance data (Fig 4). No significant groupings were identified between sample location, HIV status or primary versus permanent teeth. Samples taken from the same patient did seem to cluster together, however there was significant overlap between patients. Principal coordinate plots were also generated from both Bray-Curtis scores and weighted and unweighted UniFrac scores, but no grouping differences were identified between individual patients, HIV status or sample location including primary versus permanent teeth (Fig 5).

## Discussion

Our examination of the pediatric oral microbiome identified several significant differences or trends between adult and primary dentition, and specimen sampling site, but did not identify a difference between HIV status, dental characteristics or between individual teeth. Fluctuations in relative OTU abundance were observed between sampling sites. In particular, the relative abundance of Firmicutes appeared to increase in mucosal samples, while samples from teeth had a greater diversity of different phyla. Prior studies have also found that mucosal or saliva samples are dominated by the Firmicutes phyla, with much lower concentrations of TM7 or the Spirochaetes phylum [4]. It is unclear if properties inherent to Firmicutes contribute to its increased relative abundance in saliva and mucosal samples. Wessel et al. examined the characteristics of planktonic and biofilm-forming bacteria in tooth and saliva samples, and concluded that bacteria observed in saliva samples had lower adhesion forces compared to biofilm bacteria [35]. It is also possible that the greater diversity of highly abundant genera observed in our tooth samples are secondary to biofilm formation, which may provide a matrix permitting the adherence of a richer community of bacteria relative to mucosa samples. Like other studies, we did find a significant change in the microbial diversity between teeth samples and mucosal samples, although differences between individual teeth were not significant, and no clustering was identified between sample locations noted using NMDS analysis [5, 36]. There are also differences within the existing literature regarding bacterial abundance at different oral sites, with some studies observing differences in diversity between mucosal or saliva samples, while others have found greater diversity from plaque or tooth samples [5, 36].

Low enrollment confounded the analysis of dental characteristics, and no significant differences were appreciated in bacterial diversity associated with these dental characteristics. Periodontal disease was minimal in enrolled subjects, regardless of plaque score. The average DMFT (Decayed, Missing or Filled Teeth) score for permanent dentition was 3, for mixed dentition 0.714 and for primary dentition 3.5. The prevalence of caries was generally low; no permanent teeth had untreated caries, while 29% of mixed teeth and 50% of primary teeth had untreated caries. Previous studies have found shifts in the oral microbiota that are associated with caries formation in children, and dental plaques may progress to caries [36, 37]. Certain genera and species have been associated with the formation or avoidance of caries, but we did not observe similar results. Like other studies, our tooth samples were dominated by the genera *Actinomyces*, *Prevotella*, *Streptococcus*, *Fusobacterium*, *Leptotrichia*, *Corynebacterium*, *Veillonella*, and *Rothia*, which are all well-known oral flora and have been identified as core genera in other studies of dental plaque [5, 38]. *Gemella* and *Abiotrophia* have been found to be more abundant in dental plaques from children without caries, yet our patients with a poor plaque score had an increased abundance of *Gemella* while *Abiotrophia* was found in children with both poor and good plaque scores. (Fig 2B) [39]. Other studies have linked *Fusobacterium* to periodontal disease, which does not correlate with our finding of increased *Fusobacterium* abundance in patients with a good plaque score without evidence of periodontal disease [40]. Given the small sample collected from dental plaques and small number of patients with a poor plaque score, these differences in findings may be a sampling error. However, it emphasizes the difficulty of identifying significant changes in plaque microbiota that may be predictive of disease. For instance, changes in oral microbiota have been associated with tooth surface location, which was not examined in this dataset [5, 41] and many studies strictly control for any possible factor that may influence the oral microbiome, such as recent tooth brushing or recent meals. If next-generation sequencing technology is to be applied to the diagnosis of periodontal disease, it must be easily performed and reproducible between different healthcare providers and patients. Further investigation into fluctuations between biofilms and planktonic bacteria associated with periodontal disease is needed to determine what factors may impact on the abundance or diversity of different bacterial species and how they may relate to disease pathogenesis.

Despite limitations imposed by uneven enrollment into patient subgroups, we identified significant differences between the oral microbiota of the patients. No single patient responsible for the variation could be identified, indicating more generalized differences existed between the study subjects. Although only one patient under five years of age was enrolled, her sample had a markedly lower Shannon diversity score compared to the other samples. If additional children under five years of age had been enrolled, it is possible that a more robust difference could have been observed. These differences support previous observations of the evolution of the oral microbiome with age and dental health. It is likely additional changes would be apparent with increased enrollment of young children and a greater diversity of oral habits and dental health status.

Although a significant difference was not identified between primary and permanent teeth by ANOVA, additional investigation was performed to examine potential differences between primary and permanent teeth. The ten most abundant genera responsible for 70–80% of total abundance were examined. Most common genera were shared between primary and permanent teeth samples. Primary teeth were found to have increased abundance of *Rothia* and *Actinobacillus*, while the permanent teeth had increased abundance of *Actinomyces*, which has been previously observed as part of the natural evolution of oral flora [36, 41]. However, unlike other studies, we did not observe changes in the abundance of *Porphyromonas*, or *Tannerella* with age. Nor did we find an increased prevalence of *Lactobacillus* in our samples, as was identified in a study of plaque samples from pre-school aged children[36]. The relatively small number of primary teeth enrolled into our study may account for the lack of power to identify these changes. However, the variability observed in the literature is also likely a function of the many factors impacting observations of oral microbiota, including transient changes in flora that may be observed with sampling limited to a single point in time.

Significant changes in microbial diversity were not observed between HIV-positive and HIV-negative samples. The enrolled HIV-positive patients were generally very well-controlled with high CD4 counts and low viral loads, reflective of the current high standard of care for patients living in the United States with HIV infection. Perinatal HIV transmission declined substantially between the time the study was conceived and implemented. The proportion of pediatric HIV patients with poorly controlled disease and substantial degrees of immunosuppression likewise declined substantially, making comparison of highly immunosuppressed patients with well controlled patients more problematic than originally planned. Only three patients had a CD4+ T-cell count less than 500 cells/mm^3^ at the time of sampling, but significant changes in diversity were not observed in these patients. For example, it is well-known that some oral opportunistic infections are rarely seen in well-controlled HIV-positive patients, and we found that the oral flora of well-controlled HIV patients is essentially the same as HIV-negative patients. As the standard of clinical care for HIV has improved, identifying patients with low CD4+ T-cell counts for enrollment into studies has become challenging. Many patients with low CD4+ T-cell counts may present to medical care only when seriously ill, adding another layer of complexity into evaluation of their microbiota. Additionally, some studies have suggested that the administration of anti-retroviral medications (ARV) themselves may alter the oral microbiota, and have found that lingual samples from HIV+ patients on ARVs have decreased abundance of *Lachnospiraceae* and *Neisseria* [24, 42]. We did not observe a change in the abundance of *Neisseria* between our patient cohorts, although *Lachnospiraceae* did appear to decrease. It should be noted that *Lachnospiraceae* was a relatively low abundance species in both patient groups. It does appear that in untreated HIV positive patients, changes occur in the oral microbiota, particularly as HIV viral load increases [24]. Although our study was limited by low-enrollment of poorly controlled HIV positive subjects, a larger study examining a greater cross-section of the HIV-infected community is likely to identify significant changes in the oral microbiome.

As next-generation sequencing technology has become both more affordable and accessible, it has become more attractive as a potential tool for better diagnosis of diseases caused by shifts in microbial flora. Significant challenges must be addressed before this technology can be applied to clinical medicine. In particular, the oral microbiome appears to be a fluid and variable environment, in which a standardized measurement technique has yet to be established. Changes in the oral microbiome are readily observed in the pathogenesis of periodontal disease and caries formation, and a link to HIV oral disease such as candidiasis, necrotizing gingivitis or Kaposi’s sarcoma also exists. Ultimately, it may be possible to generate a diagnostic test predictive of a patient’s risk of oral disease to guide clinical monitoring and management. Larger and more inclusive studies with fewer sampling limitations will be needed to define the natural changes that occur as primary dentition evolves into permanent dentition, and to standardize the measurement of oral microbiome samples.
